# De Novo Genome Assemblies of Kazakh Horses: Adai, Zhabe, Mugalzhar and Naiman

**DOI:** 10.3390/ani16213374

**Published:** 2026-10-26

**Authors:** Rakhim Kanat, Yuliya Tugunova, Zagipa Sapakhova, Tatyana Ulyanova, Dinara Salimova, Ulbolsyn Kaisarova, Indira Beishova, Malika Shamekova

**Affiliations:** 1 Tanir Research Laboratory, Almaty 050040, Kazakhstan; 2 Institute of Plant Biology and Biotechnology, Almaty 050010, Kazakhstan; 3 Zhangir Khan West Kazakhstan Agrarian Technical University, Oral 090009, Kazakhstan

**Keywords:** Kazakh horse, de novo, genome assembly, genome annotation, Adai, Zhabe, Mugalzhar, Naiman

## Abstract

Kazakh horses represent an important cultural heritage of the Kazakh people, shaped over thousands of years across the territory of present-day Kazakhstan. Long-term adaptation to extreme natural conditions has led to the development of a unique set of characteristics that ensure the high survivability, ecological plasticity and economic value of these horses. Long- and short-read sequencing was performed for three breeds (Adai, Zhabe, Mugalzhar), while Naiman was sequenced exclusively with nanopore long-read data. The resulting reads were assembled, and the genomes were subsequently annotated. The assembled genomes showed high contiguity with an average contig N50 value of 9.68 Mb (9,681,711 bp) for Adai, Zhabe and Mugalzhar, while the resulting contig N50 was 17.09 Mb (17,090,216 bp) for Naiman, and average assembly length was 2.42 Gb (2,442,471,376 bp). These are individual reference assemblies that establish foundations for future comparative genomics and pangenome development. Gene annotation yielded 19,584, 19,941, 18,695 and 20,572 protein-coding genes for Adai, Zhabe, Mugalzhar and Naiman, respectively. These assemblies complement existing assemblies for future genetic analysis, research into the geographic and breed origins of domestic horses, and breed conservation efforts, and will contribute to ongoing equine pangenome efforts.

## Simple Summary

Kazakh horses represent an important cultural heritage of the Kazakh people, shaped over thousands of years across the territory of present-day Kazakhstan. In our study, we investigated the genomes of the indigenous Kazakh horses Adai, Zhabe, Mugalzhar and Naiman. Long- and short-read sequencing was performed for the genomes of these indigenous Kazakh horse types at the individual level (Adai, Zhabe, Mugalzhar and Naiman). The genomes were subsequently assembled and annotated. The assembled genomes demonstrated high coverage, with an average contig N50 value of 9,681,711 bp for Adai, Zhabe, and Mugalzhar, whereas the contig N50 value for Naiman reached 17,090,216 bp, with an average genome length of 2,442,471,376 bp. Gene annotation identified 19,584, 19,941, 18,695 and 20,572 protein-coding genes in Adai, Zhabe, Mugalzhar and Naiman, respectively. The resulting genomic data may serve as a valuable resource for future studies on the origin and divergence of horse domestication, providing continuous genome assemblies comparable to other high-quality reference genomes currently available.

## 1. Introduction

Horses (*Equus caballus*) are very important animals for many types of scientific research, especially genome assembly projects. Horses were domesticated around 5500 years ago in the Eurasian steppes. In recent decades, they have emerged as valuable models for studying evolution, genetics, and practical applications such as breeding and disease resistance. Beyond purely scientific interest, the improved equine genome has a direct impact on veterinary medicine and breeding programs [1,2]. Accurate assembly allows for better annotation of disease-associated loci, facilitates marker-assisted selection, and supports the development of genomic models for predicting traits such as speed, endurance, and disease susceptibility [3]. While the genome size is typical for mammals (2.5 Gb), its size, combined with high heterozygosity, makes de novo assembly more challenging than assembling smaller genomes from other organisms, such as flies or bacteria. However, modern sequencing technologies allow for the production of high-quality long reads, making genome assembly with a small number of contigs more accessible [4].

Since the publication of the first reference assembly of the equine genome in 2009 and its subsequent refinement to a telomere-to-telomere assembly [5], numerous breed-specific genome assemblies have become available. These include the reference Thoroughbred genome [6], telomere-to-telomere assemblies of the Mongolian horse, Debao pony, and Kazakh Zhabe horses [7], high-quality assemblies of the Shire, Arabian, Finnhorse [8], Dutch Warmblood, and Friesian horses [9], and partial assemblies of the Lipizzaner breed available through NCBI.

The Zhabe, Adai and Mugalzhar types of Kazakh horses were developed through targeted selection of the best representatives of the Zhabe type and reflect various adaptive and productive specializations [7]. They form the genetic basis for resilience to harsh climatic conditions and limited forage resources. These horses are characterized by their capacity to survive prolonged winter periods, a pronounced herd instinct, effective thermoregulation, and efficient energy metabolism during long winters [10]. The Zhabe and Mugalzhar breeds are distinguished by stable meat and milk production combined with the adaptive qualities of the local steppe horse, which is particularly important for food security and the sustainability of traditional livestock farming. The Adai type, which developed in the arid regions of Western Kazakhstan, exhibits pronounced adaptation mechanisms to hot climates, water shortages, and sparse pastures, representing a genetic reserve for resilience to arid conditions. In addition, Adai horses are widely used in horse racing, as well as in the production of meat and kumis [10,11].

**Figure 1:**
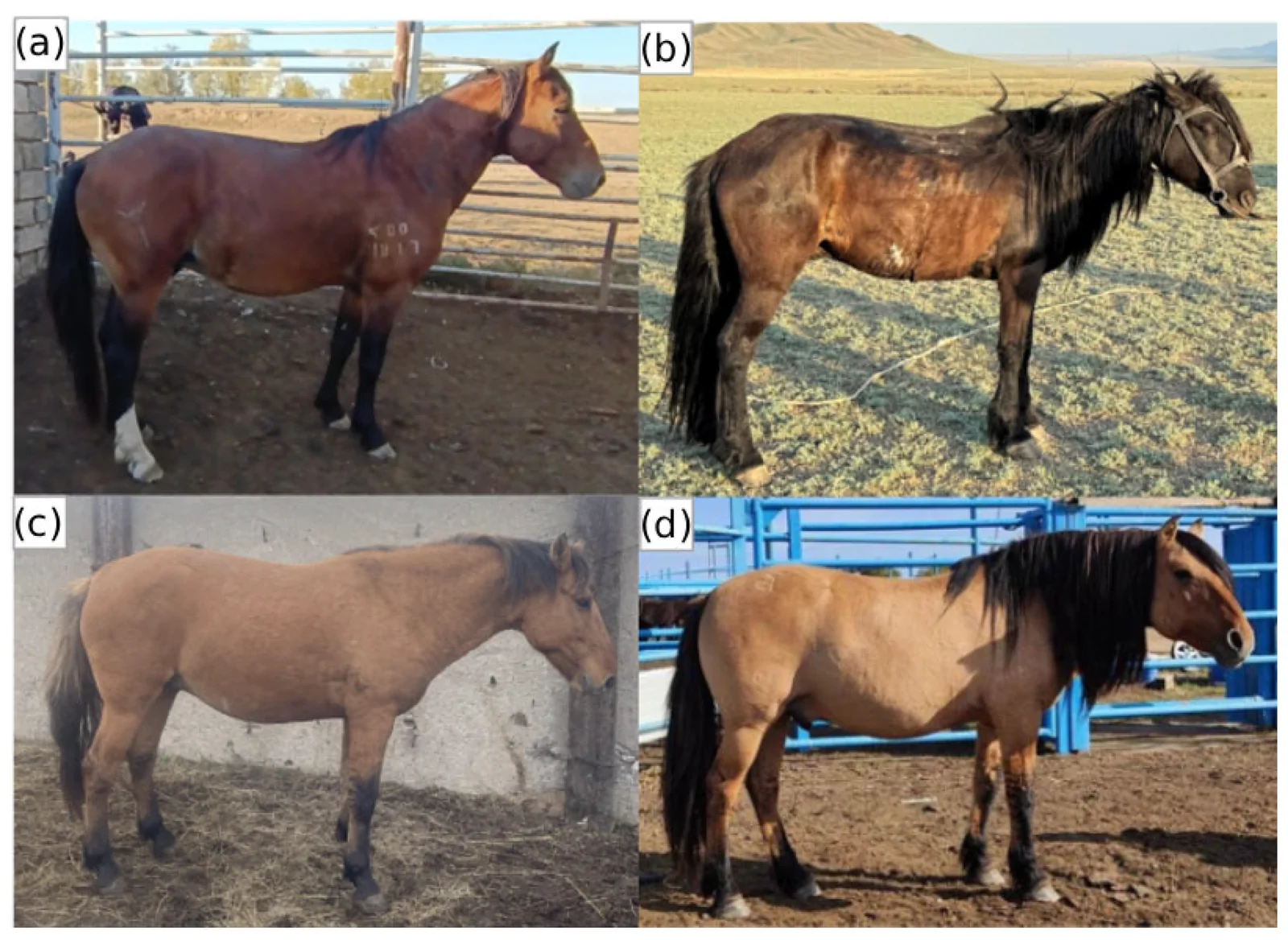
Kazakh horse breeds, (**a**) Adai, (**b**) Naiman, (**c**) Zhabe, (**d**) Mugalzhar, sequenced and assembled in this study.

In this study, both short- and long-read sequencing technologies provided by the BJI DNBSEQ platform and Oxford Nanopore Technologies (ONTs) were utilized to generate highly contiguous genome assemblies of four local Kazakh horse breeds, Adai, Zhabe, Mugalzhar and Naiman, using only long reads (Figure 1). The new assemblies, generated using high-coverage long-read sequencing, yielded highly contiguous genomes with contig N50 values of 9.68 Mb (9,681,711 bp) for the Adai, Zhabe, and Mugalzhar Kazakh horse breeds and 17.09 Mb (17,090,216 bp) for the Naiman breed, indicating overall high assembly quality. The combination of already assembled and available equine genomes may open up opportunities for further research into the origins of horses in Central Asia, selective breeding, and equine evolution by examining chromosomal rearrangements and their impact on existing patterns of distances between other horse breeds compared to local Kazakh breeds.

## 2. Materials and Methods

### 2.1. Sample Collection

Genetic material from Kazakh horses of the Adai, Zhabe, Mugalzhar and Naiman breeds for whole-genome sequencing using long and short reads was isolated from horse blood. For each breed, blood was collected from one adult stallion into EDTA tubes. The samples were stored at −80 °C. DNA from blood samples was isolated using Qiagen DNeasy Blood & Tissue Kits (Qiagen, Hilden, Germany) according to the manufacturer’s protocol. Quantitative and qualitative DNA analysis for library preparation was performed using a Nanodrop 2000 spectrophotometer (Thermo Fisher Scientific, Waltham, USA) for subsequent dilution.

### 2.2. Sequencing Data Generation

Libraries were prepared using 1 µg of total genomic DNA from blood using the V14 Ligation Sequencing Kit (SQK-LSK114) to obtain long reads. Given the absence of short-read data for hybrid assembly for the Naiman breed, we prioritized maximizing long-read coverage depth. To compensate for this and ensure the Naiman assembly reached the target depth, two libraries were prepared for this sample, whereas a single library sufficed for the Adai, Mugalzhar, and Zhabe samples. PromethION flow cells (FLO-PRO114M) with R10 series nanopores were used to sequence the generated genomic libraries (Oxford Nanopore Technologies, Oxford, England).

MinKNOW (version 25.05.14) was used for data acquisition with default parameters, except for the 48 h runtime. After 24 h, the flow cells were washed, and a second library load was completed to maximize data yield. The raw pod5 files from the PromethION device were base called using Dorado software (v0.9.6+0949eb8d) provided by Oxford Nanopore Technologies with the super accuracy (SUP) basecalling model (dna_r10.4.1_e8.2_400bps_sup@v5.0.0) and the –emit-fastq flag to generate fastq files. The resulting fastq files were evaluated with NanoPlot (v1.46.2) [12], followed by filtering and quality control with NanoFilt (2.8.0) [13] using the following parameters: minimum phred quality 15, minimum size 500 bp, and no trimming, as adapters were trimmed during base calling with Dorado.

The DNBSEQ short-read sequencing platform was used to generate fastq files. DNBSEQ short-read library preparation included DNA fragmentation, size selection, end repair and A-tail addition, adapter ligation, product size selection, PCR reaction, and library quality control according to the manufacturer’s protocol using the MGIEasy Fast FS Library Prep Set V2.0 for 150 bp paired-end sequencing. Initial filtering of raw reads was performed using SOAPnuke software (v2.1.9) (Y. Chen et al. 2018) [14], developed by BGI (Shenzhen, China), with the following parameters: -n 0.001 -l 10 -q 0.5 –adaMR 0.25 –polyX 50 –minReadLen 150. Read quality was checked using MultiQC (v1.27.1) [15]. Then, fastp (v0.23.2) [14] was used to filter out raw reads to obtain only high-quality reads with a phred score above 30.

### 2.3. Assembly

The genome size of all four Kazakh horse breeds was estimated using the k-mer histogram method with Kmergenie (v1.7016) [16] on short reads obtained from DNBSEQ sequencing results after filtering with fastp. Coverage was calculated by dividing the total number of sequenced bases in each library by the size of the assembled genome. Long reads were assembled using Flye (v2.9.6) [17], with parameters set to --nano-hq for nanopore data and genome size calculated previously by Kmergenie. Because ONT reads retain systematic insertion/deletion errors, the rough assembly was polished using medaka (v2.2.0) [18], which uses neural networks to map individual sequencing reads onto a reference sequence, yielding results superior to sequence graph-based and signal-based methods. Following long-read polishing, DNBSEQ short reads were aligned to the polished assembly using bwa2 (v0.7.19) [19], and variant calling was performed using Pilon (v1.24) [20] for two additional rounds of error correction. This hybrid polishing approach leverages the contiguity of long-read assemblies while incorporating the base-level accuracy of short-read data. Chromosome-scale scaffolding was achieved using RagTag (v2.1.0) [21] with T2T-horse1.0 as the scaffold reference. To remove contaminant sequences from the genome assemblies using the NCBI-provided [22] database, the NCBI Foreign Contamination Screen (FCS) tools (v0.5.5) (FCS-adaptor and FCS-GX) were used.

The assembled genomes were validated by mapping the original short-read (DNBSEQ) and long-read (Oxford Nanopore) datasets back to the final polished assemblies. Alignment statistics, depth of coverage, and the proportion of reads that successfully aligned were used to evaluate completeness, accuracy, and structural correctness using QUAST v5.2.0 [23] (using the official horse reference genome (TB-T2T) as a guide). The final polished assemblies were evaluated with BUSCO (benchmarking universal single-copy ortholog) v5.4.5 [24] (lineage dataset laurasiatheria_odb12 for animal genomes). Metrics reported include total assembly size, N50, number of contigs, completeness (% BUSCO single-copy orthologs), and duplication rate.

Consensus quality value (QV) was calculated using Merqury v1.4.1 [25] to estimate base-level accuracy of de novo assemblies without requiring a reference genome. Short-read DNBSEQ data was used to generate a k-mer database with meryl (k = 21), which was then compared against k-mers extracted from each assembly. Naiman genome assembly (long read only) underwent identical quality assessment using the same method derived from high-accuracy long reads. K-mer frequencies were calculated using Jellyfish v2.3.0 [26] with k = 21. Genome size estimation and heterozygosity inference were performed using GenomeScope v2.2.1 [27] with ploidy assumption *p* = 2.

EquCab3.0 was used as the alignment reference for structural variant detection (minimap2 –ax asm5 + Sniffles). It remains the community-standard equine reference genome, ensuring our SV calls are directly comparable to the existing body of equine genomics literature. Notably, EquCab3.0 does not contain a complete Y-chromosome assembly. TB-T2T was used where a gapless, telomere-to-telomere assembly is essential: (a) as the scaffold reference for RagTag chromosome-scale scaffolding; (b) as the guide reference for QUAST evaluation of structural correctness; and (c) for Y-chromosome coverage analysis, as it represents the first T2T assembly of the horse Y-chromosome and thus the only suitable benchmark for that region.

Each de novo assembly was aligned to the EquCab3.0 reference using minimap2 with the -ax asm5 preset, which is optimized for mapping highly similar assemblies while accommodating large indels and structural variations. The resulting Pairwise Mapping Format (PAF) files were converted to BAM format and sorted using SAMtools (v1.19.2) [28]. Structural variants were subsequently identified using Sniffles (v2.7.3) [29], a long-read SV caller designed to detect complex rearrangements from assembly alignments. A minimum support threshold of 3 reads (or assembly segments) and a minimum variant size of 50 bp were applied to ensure high-confidence calls. Variant types classified by Sniffles included DEL (deletion), INS (insertion), INV (inversion), DUP (duplication) and VCF files filtered for PASS variants only, low-support calls (<3 reads) excluded.

Mitochondrial genomes were extracted from raw reads using MitoHiFi [30] using ‘*Equus caballus*’ flag and min length of 14,000. All 37 mitochondrial genes for all four samples were identified and assembled into circular contigs.

### 2.4. Annotation

The de novo assembled genomes (final polished FASTA file) were subjected to a two-step processing: (i) identification and masking of repetitive sequences in de novo assembled genomes, followed by (ii) structural and functional annotation of protein-coding genes, non-coding RNAs and other genomic features.

A custom library of repeat sequences was created using RepeatModeler2 (v2.0.2) [31]. The genome was first indexed (BuildDatabase) and then analyzed with the -LTRStruct option to activate the LTR-retriever module, which ensures accurate detection of long terminal repeats. The de novo library was then passed to RepeatMasker (v4.1.5) [32] for soft masking of the genome using the following flags (–xsmall). Statistics (overall percentage and types) were obtained using calcDivergenceFromAlign.pl.

For structural annotation, Braker2 pipeline [33] was chosen, which uses GeneMark-ETP [34] and AUGUSTUS [35] for gene prediction. The workflow included external evidence in the form of RNA-seq data, as well as proteins from closely related species, namely, Przewalski’s horse (GCF_000696695.1_Burgud_protein.faa), donkey (GCF_041296235.1_EquAss-T2T_v2_protein.faa), and the mammalia_odb10 2 protein databases. The pipeline works by training GeneMark-ETP on aligned RNA-seq data and protein databases from closely related species, then training AUGUSTUS for gene prediction on the same external data together with the GeneMark-ETP results, and finally combining the results with TSEBRA [36].

Genomic annotation was performed using reference sequences retrieved from the European Nucleotide Archive (ENA) under BioProject accession number PRJEB53382. These sequences represented a comprehensive allele-specific expression resource for the equine transcriptome [37]. This dataset comprises Illumina paired-end RNA-seq reads (2 × 150 bp) from multiple tissues (102) from two adult Thoroughbred mares, with 526 Gb size. While these data are not breed-matched to our Kazakh horses, they provide comprehensive splice junction information across diverse equine tissues, enabling accurate exon–intron boundary prediction. The tissue diversity of the reference RNA-seq compensates for the lack of breed-specific transcriptomes.

Paired-end Illumina RNA-seq reads (2 × 150 bp, 150× genome coverage) were trimmed with fastp (v0.23.2) and aligned to the masked genome using STAR (v2.7.11a) [38]. The sorted BAM file provided splice junction data for BRAKER2 [39].

All predicted proteins were processed with AGAT and InterProScan (v5.65-104.0) [40] using the default member databases (Pfam, SMART, CDD, TIGRFAMs, PROSITE, Gene3D, PANTHER) and models were excluded when they lacked both RNA-seq splice-junction support and a detectable protein domain. Redundant models—loci with overlapping models or excessive similarity indicative of assembly duplication—were flagged via MAKER2 using the Annotation Edit Distance (AED), retaining the model with the best transcriptomic and proteomic support.

To assess the quality of the annotations, BUSCO v5.6.2 was used in protein mode, using the db (laurasitheria_odb12) function to determine BUSCO completeness metrics. The Annotation Edit Distance (AED) distribution was calculated using MAKER2 to ensure high consistency with RNA-seq and protein data. Gene counts were obtained from BRAKER2 + MAKER2 [41] and compared to reference genomes of the same equine species. All visualizations were conducted in R (v4.6.1) (Ihaka and Gentleman, 1996) using tidyverse [42] and its ggplot2 package [43].

## 3. Results and Discussion

### 3.1. De Novo Assembly of Horse Genome

Across the three hybrid assemblies (Adai, Zhabe, Mugalzhar), an average of 153.15 Gb of data was generated per sample, comprising 96.10 Gb of short reads and 57.05 Gb of long reads (estimated at ~61× average haploid coverage across hybrid assemblies; Naiman at 47×) (Table 1). The calculated contigs N50 for long reads were 12,921, 17,354, 11,833 and 16,739 bp for Adai, Zhabe, Mugalzhar and Naiman, respectively, with an average of 14,712 bp. Short-read sequencing yielded approximately 30× haploid coverage for all genomes, with the exception of the Naiman breed, which was sequenced solely using nanopore reads. Quality control metrics revealed that for every sample, over 98% of reads possessed a Phred score greater than 20, while 95% exceeded a score of 30. Average GC (%) content was 43.59, 43.16 and 42.63 for Adai, Zhabe and Mugalzhar, respectively. Detailed statistics on reads obtained from both sequencing technologies are presented in Table 1 for each sample individually.

The comparison between our hybrid assemblies (Adai, Zhabe, Mugalzhar) and the long-read-only assembly (Naiman) demonstrates that sufficient long-read coverage can compensate for absent short-read data. Despite lacking Illumina data, Naiman achieved superior contiguity (contig N50 = 17.1 Mb vs. 9.68 Mb average for hybrids) and a maximum contig size of 62.3 Mb, suggesting that deep nanopore sequencing alone may suffice for reference-quality equine assemblies in resource-limited settings. This finding is particularly relevant for future conservation genomics of endangered or remote horse breeds.

**Figure 2:**
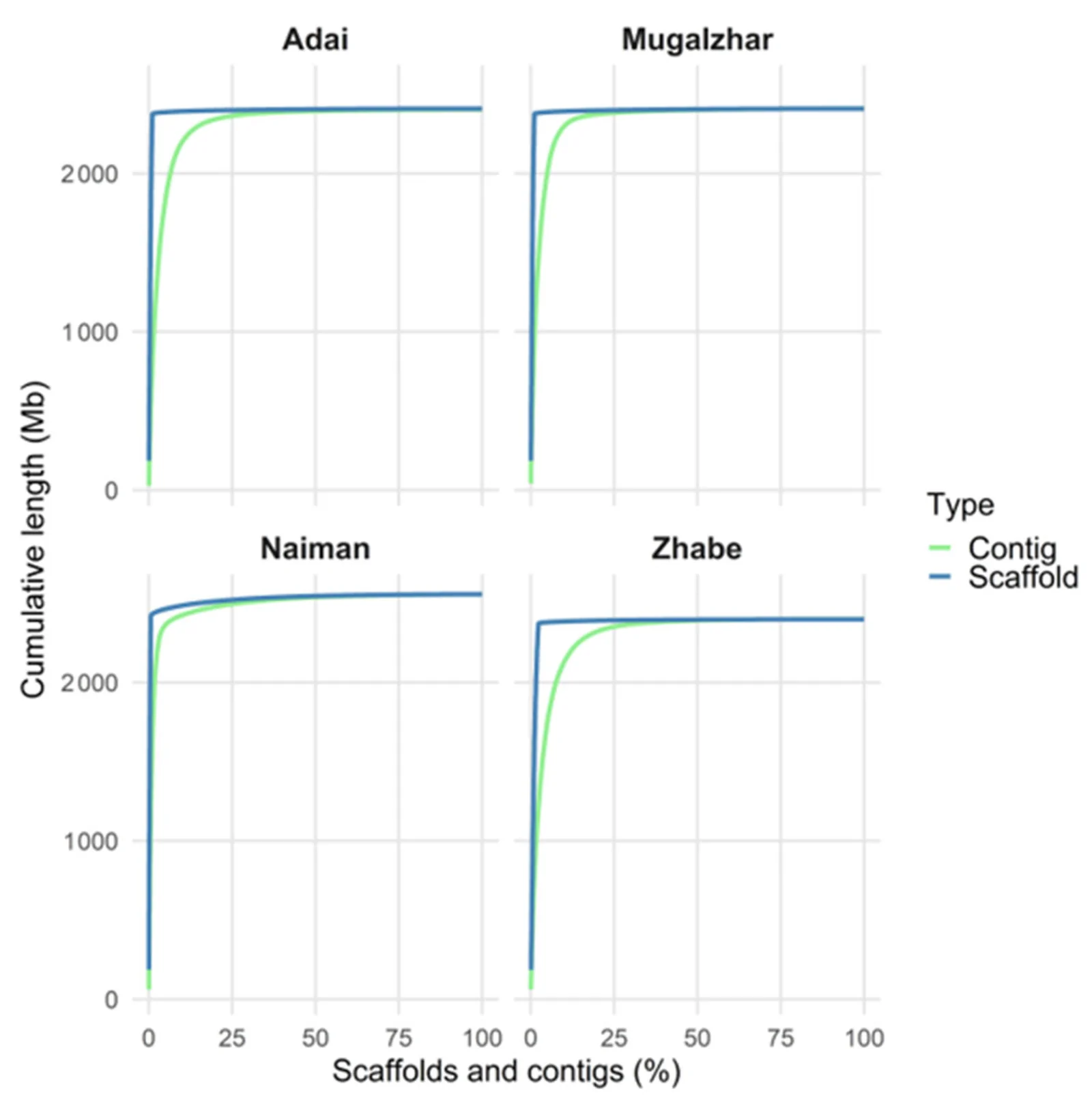
Distribution of the cumulative scaffold length of Kazakh horse genome assemblies of Adai, Mugalzhar, Zhabe, and Naiman.

**Figure 3:**
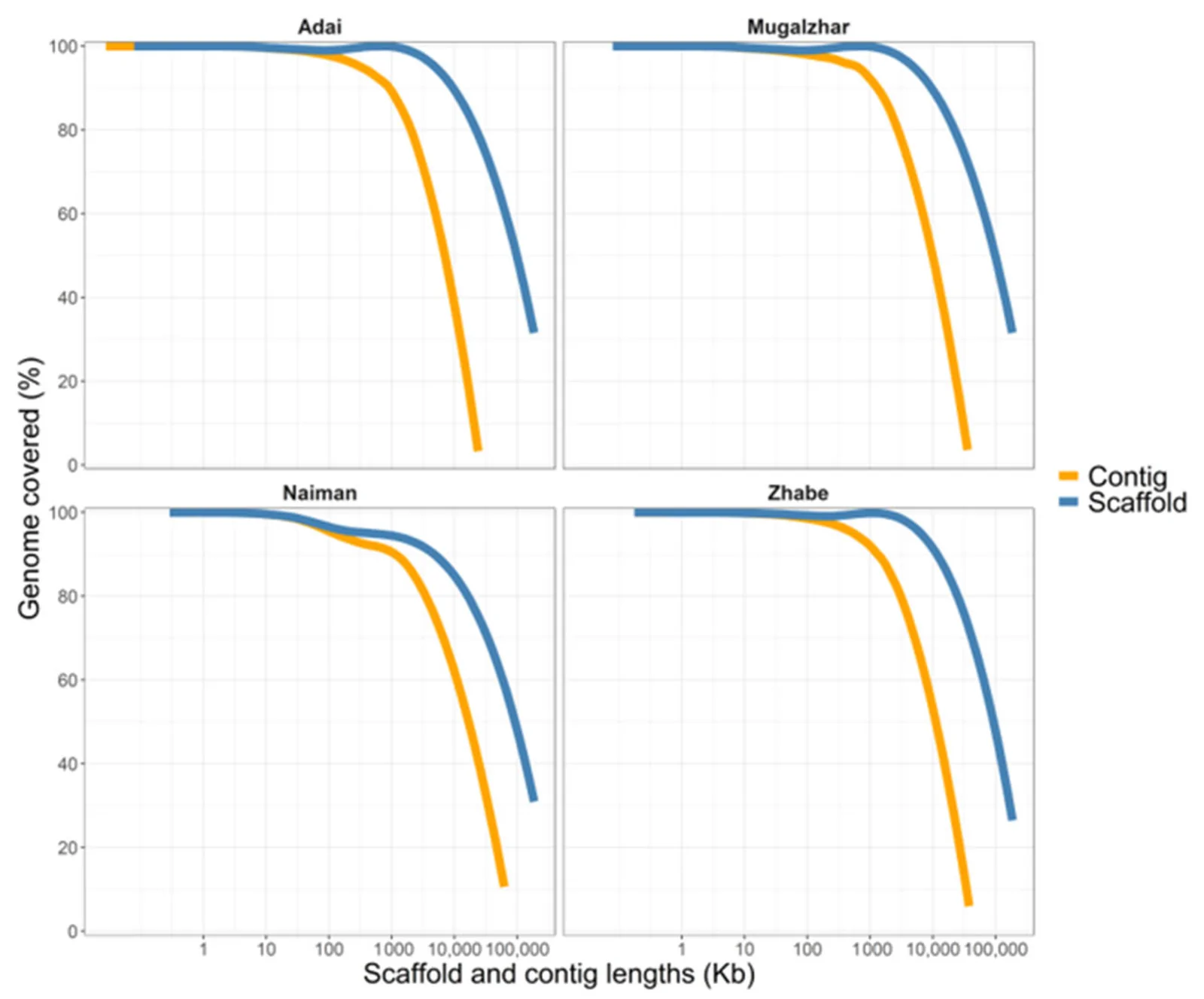
The percentage of the genome covered by contigs of a given length or longer. The X-axis represents contig lengths. For a given length Y, the Y-axis shows the fraction of the genome covered by contigs of length Y or longer.

The assemblies of Adai, Zhabe, Mugalzhar, and Naiman consisted of 4723, 2650, 4356, and 6248 contigs, respectively, with contig N50 values of 7.17, 11.59, 10.28, and 17.09 Mb. All assemblies had a total length of approximately 2.5 Gb (Table 2). These metrics correspond to previously published assemblies. All assemblies exhibited high contiguity, as reflected by their N50 values, with the 1000 longest contigs accounting for more than 80–90% of the total assembly length in all genomes. In the Naiman assembly, the 100 longest contigs captured a comparable proportion (Figure 2 and Figure 3, and Supplementary Table S3). We also compared our assemblies on de novo genome assembly of the Kazakh native horse Zhabe done on long read sequencers by Assanbayev et al. in terms of assembly metrics (Supplementary Table S4).

The completeness and accuracy of our de novo assemblies were assessed in several ways, comparing them with independent mapping data from the original short raw reads, with independently sequenced mRNA data provided by Heath et al. available at ENA under accession number PRJEB53382, and with an alternative reference genome assembly of the same species, available at NCBI under accession number PRJEB53382. All reads to this newly assembled genome were realigned, and 99% of the entire genome showed a minimum coverage of 18× long reads and 38× Illumina reads for Adai (long, short), Zhabe (long, short), Mugalzhar (long, short), and Naiman (long) (Supplementary Table S1).

K-mer-based consensus quality assessment revealed distinct accuracy profiles between the short-read and long-read assemblies. The hybrid assembly achieved a QV of 42.31, 41.78, and 41.88 for Adai, Mugalzhar, and Zhabe, respectively, while the long-read assembly of the Naiman genome attained a QV of 33.74. GenomeScope2 modeling revealed distinct heterozygosity profiles across the four assemblies. The estimated heterozygosity rates were 0.41%, 0.34%, 0.45% and 1.21% for Adai, Zhabe, Mugalzhar and Naiman, respectively.

We note that reference-guided scaffolding inherently introduces a degree of reference bias, as contig placement and orientation are constrained by homology to T2T-horse1. However, this does not affect sequence content: the contig-level assemblies remain fully de novo, and any scaffold junction can be traced through the AGP records. Regions that could not be placed or oriented on the reference chromosomes are retained as unplaced scaffolds rather than discarded, preserving the individual-level genomic feature sequence (~5% of each assembly did not align to TB-T2T, largely corresponding to structural variants). Consequently, analyses that are sensitive to adjacency assumptions—notably breakpoint localization of structural variants—should ideally be interpreted at the contig level, whereas chromosome-scale scaffolds are intended to facilitate comparative visualization and coordinate-based queries.

Completeness of the genome was assessed using BUSCO on database laurasiatheria_odb12, and showed high coverage (Table 3). To further evaluate assembly quality, all reads were mapped to the assembled genome, and the assembled contigs were aligned to the reference genome, achieving 99% coverage. The alignments of reads to the assembled genomes showed very high coverage consisting of 99.8%, 99.9%, 99.8% and 99.8% for Adai, Mugalzhar, Zhabe and Naiman breeds, respectively. The genome alignment of our assembly with TB-T2T showed high synteny between the assemblies. Quast-lg analysis showed that 94.99%, 95.47%, 95.29% and 95.74% of the assembled genomes of Adai, Mugalzhar, Zhabe and Naiman breeds mapped to the TB-T2T genome assembly, covering a total alignment length of 2,385,091,973, 2,390,440,687, 2,449,999,574 and 2,382,530,715 bp. A total of 16,753,994, 17,201,171, 16,314,348 and 60,192,839 bp were unaligned, or 212, 190, 114 and 169 contigs could not be aligned to the TB-T2T genome, and 1008, 1060, 561 and 2680 contigs of the Adai, Mugalzhar, Zhabe, and Naiman breeds respectively could only be partially aligned (not full length of contig aligned). The remaining ~5% may represent genuine structural divergence, individual-level genomic feature sequences, repetitive regions, or assembly artifacts. While we attribute most discrepancies to structural variation, alternative explanations cannot be ruled out without orthogonal validation. Aligned and misaligned contigs could result from structural variations between our genome and the EquCab3.0 reference genome, such as large insertions/deletions, as well as repetitive regions and/or alternative haplotypes causing assembly errors. To characterize the nature of these discrepancies, we analyzed the alignments using Sniffles, which revealed substantial structural variation across the four breeds. Specifically, we identified approximately 17,000 deletions, 15,000 insertions, 1300 inversions, and 30 duplications relative to the reference genome. These results confirm that the unaligned and partially aligned contigs are largely attributable to large-scale structural variants (particularly insertions and deletions) and complex rearrangements. A detailed breakdown of these variants by breed is provided in Supplementary Table S2.

Importantly, given that these assemblies derive from single individuals, observed differences from TB-T2T should be interpreted as individual-level variation rather than breed-defining structural variants. Some variants may reflect haplotypic differences, repetitive region assembly challenges, or reference bias rather than true breed-specific genomic architecture. Future pangenome studies with multiple individuals per breed will be required to distinguish population-level from individual-level structural variation.

Next-generation sequencing technologies, particularly long-read sequencing methods, have significantly increased the availability of genome assemblies, producing high-contiguity assemblies using nanopore sequencing alone, as demonstrated by the Naiman breed genome assembly presented in this study, which achieved a contig N50 of 17.09 Mb. Although the lack of short-read data in the Naiman breed led to a greater number of final contigs than in assemblies that combined short- and long-read sequencing, the higher long-read sequencing coverage produced a more contiguous assembly, as reflected by a higher contig N50 and a larger proportion of the genome being represented by fewer contigs. In addition, post-processing of the assembly using deep learning technologies such as Medaka also improved per base genome quality [18]. Genomic assemblies incorporating long reads demonstrate several-fold higher contiguity and N50 contigs, as well as significantly fewer final contigs, compared to assemblies using only short reads [44]. In this study, the advantages of the length and contiguous reads of long-read sequencers, as well as the read accuracy of short-read sequencers, were used to assemble Kazakh horse genomes of high quality.

Among our four Kazakh horse assemblies, Naiman stands out with the highest contiguity despite lacking short-read polish, demonstrating that deep nanopore coverage (47× estimated) can produce reference-quality assemblies without hybrid approaches. The other three breeds show comparable completeness with lower long-read coverage but benefit from short-read correction at the base-pair level (BUSCO >99.3%). These trade-offs provide practical guidance for future breeding programs: when resources are constrained, prioritize long-read depth; when maximum base accuracy is required, combine both technologies.

To assess the completeness of the Y-chromosome, we aligned the Y-chromosome contigs from each assembly to the telomere-to-telomere (T2T) Y-chromosome sequence of the Mongolian horse (T2T-horse1.0) using minimap2. The Mongolian horse genome was chosen as the alignment target in place of the standard reference, as it represents the first telomere-to-telomere (T2T) assembly of the horse Y-chromosome, offering a complete and gapless benchmark, while the reference genome lacks Y chromosome sequence. The percentage of the reference Y-chromosome covered by our assemblies was calculated as the ratio of aligned bases to the total reference length. Assemblies covered 93.74% (Mugalzhar), 99.64% (Zhabe), 99.92% (Adai), and 99.98% (Naiman) of the T2T-horse1.0 Y-chromosome sequence. These results demonstrate that our long-read assemblies have potential to be used in more accurate analysis of the origin and domestication of horses [45]. These high-quality Y-chromosome sequences will facilitate future phylogenetic analyses and comparisons with other breeds.

A principal limitation of this study is the use of single individuals per breed. While this provides high-quality reference assemblies for the Kazakh horse gene pool, it does not capture within-breed genetic diversity or allele frequency distributions. Consequently, claims of ‘breed-specific’ sequences should be understood as ‘observed in the sampled individual’ rather than diagnostic of the broader breed population. Population-scale sequencing across multiple individuals per breed will be necessary to quantify within-breed diversity and validate breed-defining variants. Nevertheless, these assemblies provide essential foundational references for future pangenome studies and conservation genomics of Central Asian horse breeds.

Mitochondrial genomes were extracted from raw reads using MitoHiFi with the ‘*Equus caballus*’ flag and minimum length threshold of 14,000 bp. All 37 mitochondrial genes were assembled into circular contigs for each of the four samples with contig lengths of 16,632, 16,623, 16,602 and 16,624 for Adai, Mugalzhar, Naiman and Zhabe, respectively. Mitochondrial genes were annotated using MitoAnnotator, showing standard vertebrate mitochondrial gene order. Accession numbers for mitochondrial genomes are included in the Data Availability Statement. These mitogenomes will support future phylogenetic analyses of maternal lineages in Central Asian horse breeds.

### 3.2. Genome Annotation

Further, annotated polished assembled contigs were searched for repeats and genes. Total repeats of the assembled genomes accounted on average for 34.35% of the total assembled genome (Table 4).

AGAT (v2.0.0) and InterProScan (v5.77-108.0) were used to verify the annotation. The statistics from AGAT software are similar for all four files. On average 19,698 genes were predicted. The average number of exons per transcript is between 10 and 11, compared to 14.9 for the reference. The number of single-exon genes is between 10 and 12% of the total number of genes found, compared to 9% for the reference (Table 5).

The predicted number of mRNAs (24,745–27,486) is substantially lower than reported for EquCab3.0 (60,750) and TB-T2T (97,886). This discrepancy is primarily attributable to differences in annotation strategy rather than absence of biological content. Reference annotations aggregate transcript evidence accumulated over decades from multiple individuals, tissues and developmental stages, and each locus is typically represented by numerous transcript isoforms, inflating the total mRNA count. In contrast, our BRAKER3-based annotation was trained on a single RNA-seq resource (PRJEB53382) and a defined set of related-species proteins, and was further consolidated by filtration, which retained only the best-supported model per locus (described below). The higher number of predicted genes relative to mRNA therefore reflects a conservative, non-redundant gene set built from a narrower evidence base. Consistent with this, BUSCO assessment in protein mode recovered 91.3–94.0% complete genes after filtration, indicating that conserved single-copy orthologs are well represented.

The slightly higher proportion of single-exon genes in our assemblies (10–12% vs. ~9% in the references) can be explained by both technical and biological factors. First, where splice-junction support was absent in the RNA-seq evidence, BRAKER3 may report a single-exon model rather than a fragmented or multi-exon model. Second, our filtration pipeline retained shorter, compact gene models that longer-running manual curation efforts in reference genomes would have merged with longer transcripts or removed as artifacts. Third, single-exon genes are enriched among recent retro-duplicated genes (retrogenes), whose abundance varies between lineages and can plausibly differ between local Kazakh breeds and the Thoroughbred-derived references. We acknowledge that a subset of predicted single-exon genes may represent over-prediction, and future integration of additional tissue-specific transcriptomic data will help distinguish genuine single-exon genes from annotation artifacts.

Final gene counts varied across assemblies (18,695–20,572 genes), likely due to a combination of biological and technical factors. These include breed-specific or individual-specific gene content, differences in assembly completeness that affect gene prediction, and variation in repeat masking efficiency, particularly in repeat-rich genomic regions.

**Figure 4:**
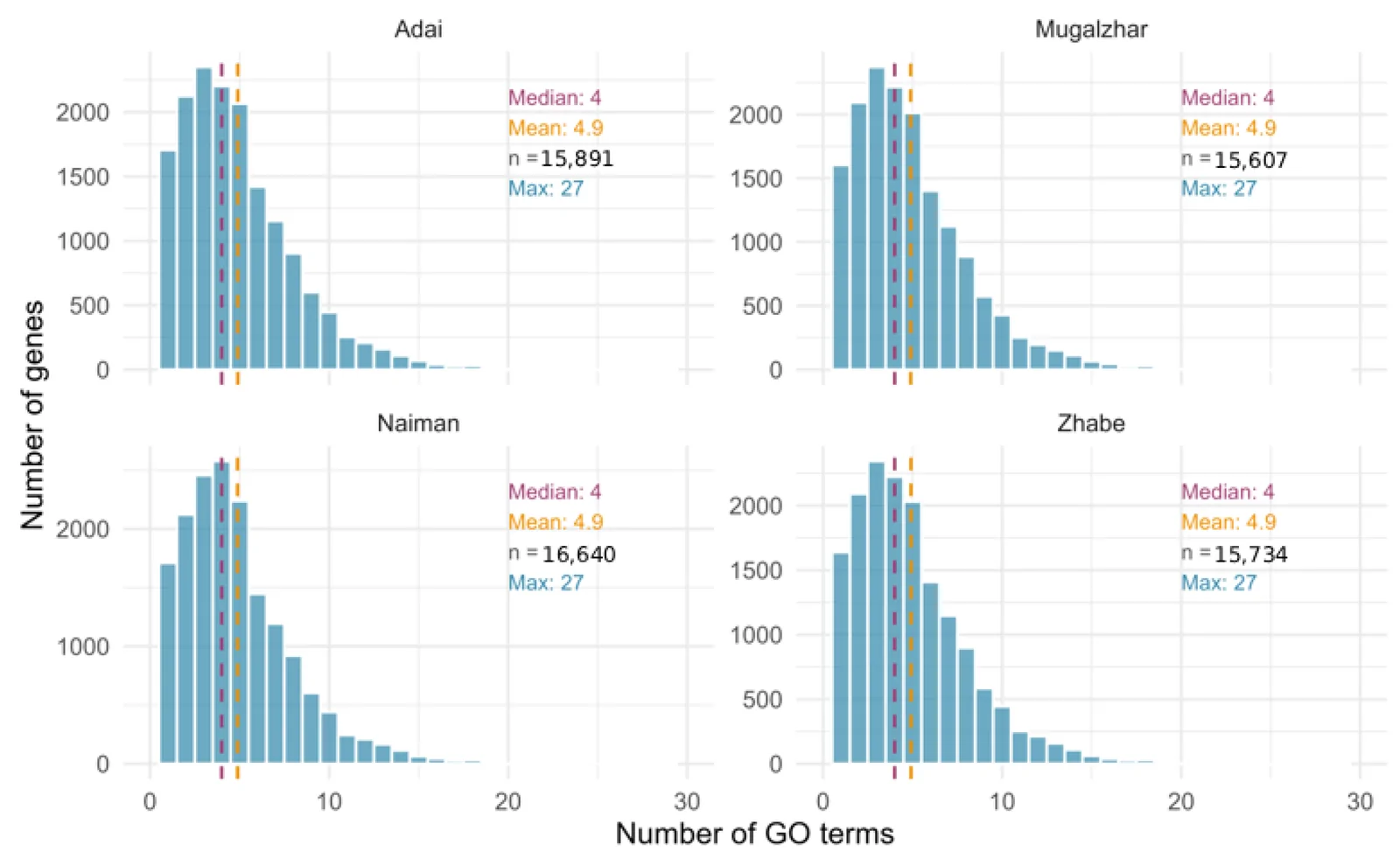
Distribution of the number of GO terms. Sequence counts: Adai (26,006 proteins), Zhabe (24,745), Mugalzhar (26,851), and Naiman (27,486). Multiple transcript isoforms per gene were included in the analysis, accounting for the difference between gene counts and transcript counts reported in Table 5.

According to the results from InterProScan, at least 91% of all proteins have functional domains. Protein sequences were filtered by low *p*-values (1 × 10^−5^) and the presence of an assigned domain from InterProScan databases (except COILS and MobiDB). Interproscan was run with the -goterms flag, which provided a comparison with gene ontology (GO). Distribution of GO terms by unique protein sequences is shown in Figure 4. All predicted transcript isoforms were included in the InterProScan analysis (Adai: 26,006 proteins; Zhabe: 24,745; Mugalzhar: 26,851; Naiman: 27,486), resulting in a median of four GO terms per protein with no extreme tail of high-annotation outliers. The distribution is similar in all four graphs, which indicates the stability of the annotation. The correctness of the annotation and the absence of redundancy are confirmed by the fact that the median value is four GO terms, as well as the absence of a long ‘tail’ on the right, i.e., the absence of proteins with a large number of assigned terms.

Based on BUSCO benchmarking performed in protein mode with laurasiatheria_odb12 (1 July 2025) database, the assemblies achieved an average completeness score of 93% and an average duplication rate of 7.4%. Before filtering, BUSCO duplication rates averaged 21–24% across breeds (Table 6), likely reflecting both true biological duplication and assembly artifacts such as haplotypic collapse or fragmented gene models. To generate a conservative, non-redundant gene set, we applied the following filtration criteria: remove genes with ≥95% sequence identity to other predicted genes within the same assembly, retain the longer isoform when multiple transcripts mapped to the same genomic locus, exclude genes with AED > 0.5 (poor agreement with RNA-seq/protein evidence). Individual statistics of BUSCO assessment are available in Table 6.

Assembly completeness can also be assessed by evaluating the extent to which known gene sequences align to the assembled genome. Modern annotation pipelines rely on RNA-Seq data as primary evidence to train gene prediction algorithms and define exon–intron structures. Publicly available RNA-Seq mRNA sequences were aligned to the four assemblies, using spliced alignment mapping tools. RNA-seq data were aligned to the predicted gene models using STAR to assess gene expression. On average, 94.99% of the predicted genes were expressed across samples, with expression rates of 96.45% for Adai, 94.37% for Zhabe, 93.58% for Mugalzhar, and 95.54% for Naiman. A high mapping rate indicates that the assembly contains continuous sequences necessary for gene model reconstruction. While transcript support strengthens confidence in predicted gene structures, it does not independently verify complete structural or functional correctness. Additional validation via proteomics or manual curation would strengthen these claims [46].

## 4. Conclusions

In conclusion, the genome assemblies of four Kazakh horse breeds—Adai, Naiman, Zhabe, and Mugalzhar—demonstrate high continuity and provide individual reference assemblies that establish a foundation for future comparative genomics and conservation efforts. These assemblies were generated using long- and short-read sequencing technologies, with the Naiman assembly relying exclusively on nanopore long reads. The contiguity metrics compare favorably with existing equine reference genomes, including an average contig N50 of 9.68 Mb for the hybrid assemblies (Adai, Zhabe, Mugalzhar) and 17.09 Mb for the Naiman long-read-only assembly. While these assemblies are based on single individuals and do not capture within-breed genetic variation, they capture individual-level genomic sequences and structural variants that distinguish Kazakh horses from the TB-T2T reference. Approximately 5% of each assembly remained unmapped to the TB-T2T reference, largely attributable to structural variants including insertions, deletions, and inversions. Additionally, near-complete Y-chromosome coverage (93.74–99.98% relative to T2T-horse1.0) enables future phylogenetic analysis of paternal lineages relevant to Central Asian horse domestication. Integrating these assemblies with existing equine genomes will expand the pangenome resource for studies on horse origins in Central Asia, selective breeding programs, and evolutionary analyses involving chromosomal rearrangements and their impact on local Kazakh breeds. These resources are made publicly available through NCBI SRA (BioProject PRJNA1425729) and Zenodo (DOI: 10.5281/zenodo.18681010).

## Figures and Tables

**Table 1:** Statistics of raw reads for each horse breed using long- and short-read sequencers. Estimated haploid coverage calculated as (Total bases ÷ assembled haploid genome size).

	Adai	Zhabe	Mugalzhar	Naiman
Total reads (long)	6,583,603	4,902,561	9,128,134	14,529,034
Total bases (long), bp	49,840,048,407	45,150,101,120	76,171,070,490	116,361,254,867
Reads N50	12,921	17,354	11,833	16,739
Total reads (short)	324,162,647	324,170,258	312,631,334	NA *
Total bases (short), bp	97,248,794,100	97,251,077,400	93,789,400,200	NA *
Estimated coverage	60×	57×	68×	47×

* NA indicates absence of short reads for Naiman breed.

**Table 2:** Quality metrics for our assemblies compared to those of other studies.

	Adai	Zhabe	Mugalzhar	Naiman	EquCab3.0	TB-T2T
Total length, bp	2,403,312,432	2,400,330,836	2,409,181,818	2,557,060,416	2,506,949,475	2,769,438,211
No. of contigs	4723	2650	4356	6248	10,986	629
Largest contig size, bp	28,530,806	61,565,570	42,276,346	62,310,566	NA	NA
Scaffold N50	91,750,146	90,428,060	91,750,146	88,921,671	87,230,776	99,223,387
Contig N50	7,170,660	11,591,424	10,283,049	17,090,216	1,502,753	99,223,387
Contig N90	866,658	1,307,088	1,466,597	1,232,442	NA	NA
Contigs L50	11	11	11	41	462	12
GC content (%)	41.51	41.45	41.52	42.30	41.5	43
No. N’s per 100 kbp	5.95	5.95	5.15	4.54	376.37	35.72

**Table 3:** BUSCO assessment results of the sequencing data.

	Adai	Zhabe	Mugalzhar	Naiman	EquCab3.0	TB-T2T
Complete BUSCO (%)	99.3	99.3	99.3	99.3	99.1	98.8
Complete and single-copy BUSCOs (%)	98.7	98.7	98.7	98.6	98.5	97.6
Complete and duplicated BUSCOs (%)	0.6	0.6	0.6	0.6	0.6	1.1
Fragmented BUSCOs (%)	0.3	0.3	0.3	0.3	0.5	0.5
Missing BUSCOs (%)	0.4	0.4	0.4	0.5	0.5	0.8

**Table 4:** Repeat content and type of the polished repeat masked assemblies.

	Adai	Zhabe	Mugalzhar	Naiman
Total repeat (%)	35.16	33.57	33.57	35.09
LINEs (long interspersed nuclear elements) (%)	14.77	15.44	14.97	15.13
SINEs (short interspersed nuclear elements) (%)	1.91	2.62	1.71	1.79
LTR retrotransposons (%)	3.16	3.99	2.06	2.44

**Table 5:** Results of annotation of genomes.

	Adai	Mugalzhar	Zhabe	Naiman	EquCab3.0	TB-T2T
Number of genes	19,584	19,941	18,695	20,572	21,426	21,772
Number of mRNA	26,006	26,851	24,745	27,486	60,750	97,886
Mean exon number per mRNA	10.9	11.1	10.9	10.8	10.2	14.9
Single exon genes	2243	2166	2243	2626	2022	1997

**Table 6:** BUSCO assessment of gene annotation before and after filtration, by removing duplicate sequences.

Breed	Complete, %	Single-Copy, %	Duplicate, %	Fragmented, %
		Before filtration		
Adai	93.0	72.0	21.0	1.0
Mugalzhar	94.0	70.0	24.0	0.9
Naiman	93.8	70.2	23.6	1.0
Zhabe	91.3	70.0	21.4	1.1
		After filtration		
Adai	92.9	85.0	8.0	1.0
Mugalzhar	94.0	86.4	7.6	0.9
Naiman	93.8	86.3	7.5	1.0
Zhabe	91.3	84.8	6.5	1.1

## Data Availability

Illumina and ONT raw reads are deposited in the NCBI SRA under BioProject PRJNA1425729. The final repeat-masked assemblies (FASTA) (adai.fasta.gz, mugalzhar.fasta.gz, naiman.fasta.gz, zhabe.fasta.gz), GFF3 annotations (adai.gff3, mugalzhar.gff3, naiman.gff3, zhabe.gff3) and mitochondrial genomes (adai.mt.fa, mugalzhar.mt.fa, naiman.mt.fa, zhabe.mt.fa) are available at Zenodo (105281/zenodo.18681010, https://zenodo.org/records/18681010, 23 October 2026).

## Supplementary Materials

The following supporting information can be downloaded at https://www.mdpi.com/article/10.3390/ani16213374/s1, Figure S1: Overview of the genome assembly and annotation pipeline; Table S1: Read alignment results of raw reads to assembled genomes for hybrid assemblies; Table S2: Number of structural variants found using Sniffles on four assembled genomes relative to the reference genome EquCab3.0; Table S3: Genome coverage of contigs sized 10, 100, and 1000 based on contig-level and scaffold-level assemblies; Table S4: Comparison with Zhabe assemblies.
