# Supplementary material for: De Novo Genome Assemblies of Kazakh Horses: Adai, Zhabe, Mugalzhar and Naiman
Source: Animals (Basel). 2026 Oct 26;16(21):3374. doi: 10.3390/ani16213374 (PMC13762031; doi:10.3390/ani16213374)
Supplement: Supplementary file 1 [file animals-16-03374-s001.zip › Supplementary Figure S1.pdf]

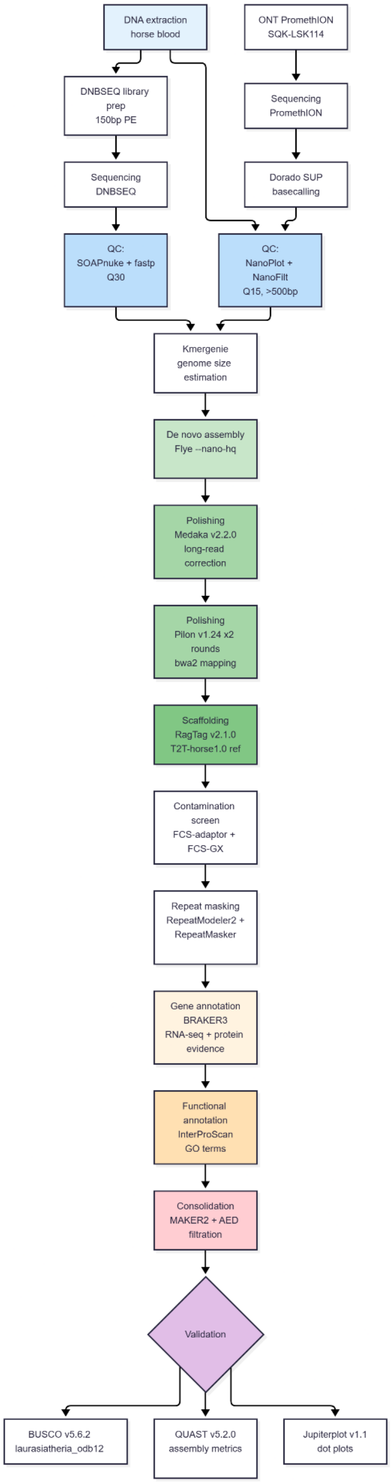

Figure S1. Overview of the genome assembly and annotation pipeline. The workflow integrates long- and short-read sequencing (de novo assembly with Flye), polishing (Medaka + Pilon), scaffolding (RagTag), and gene annotation (BRAKER3 + InterProScan) followed by consolidation and validation (BUSCO, QUAST, Jupiterplot).
