# Supplementary material for: De Novo Genome Assemblies of Kazakh Horses: Adai, Zhabe, Mugalzhar and Naiman
Source: Animals (Basel). 2026 Oct 26;16(21):3374. doi: 10.3390/ani16213374 (PMC13762031; doi:10.3390/ani16213374)
Supplement: Supplementary file 1 [file animals-16-03374-s001.zip › Supplementary Table S1–S4.pdf]

Table S1. Read alignment results of raw reads to assembled genomes for hybrid assemblies.

	Adai	Zhabe	Mugalzhar
Total raw reads	159,306,832	161,991,056	152,368,586
Reads mapped	159,055,043	161,744,538	152,201,277
Reads unmapped	251,789	246,518	167,309
Mapping rate	99.8%	99.8%	99.9%

Table S2. Number of structural variants found using Sniffle on four assembled genomes relative to the reference genome EquCab3.0.

	Adai	Zhabe	Mugalzhar	Naiman
Deletions	17,235	18,528	16,119	16,677
Insertions	14,702	16,861	14,107	14,450
Inversions	129	117	113	100
Duplications	25	31	26	26

Table S3. Genome coverage of contigs sized 10, 100, and 1,000 based on contig-level and scaffold-level assemblies.

	Adai		Mugalzhar		Zhabe		Naiman	
	Contig	Scaffold	Contig	Scaffold	Contig	Scaffold	Contig	Scaffold
Total number	4723	2924	4356	2924	2650	1414	6256	5095
Top 10, %	10.2	47.2	13.5	47.2	15.2	47.2	18.6	44.9
Top 100, %	50.8	99.0	60.6	99.0	64.8	99.3	76.4	95.6

Top 1000, %	97.7	99.7	98.8	99.7	99.2	100.0	96.3	98.2

Table S4. Comparison with Zhabe assemblies.

Metric	Adai	Zhabe	Mugalzhar	Naiman	Z1	Z2	Z3	Z4	Z5	Z6
Number of contigs	4,723	2,650	4,356	6,248	4,210	3,459	4,405	4,662	4,247	4,180
Contig N50 (bp)	7,170,660	11,591,424	10,283,049	17,090,216	25,995,087	26,306,096	25,978,232	25,961,887	19,984,809	26,032,890
Largest contig (bp)	28,530,806	61,565,570	42,276,346	62,310,566	75,750,993	67,042,342	89,046,621	62,359,761	92,322,303	63,939,966
Total length (bp)	2,403,312,432	2,400,330,836	2,409,181,818	2,557,060,416	2,551,255,215	2,534,155,455	2,590,139,805	2,602,523,862	2,608,619,890	2,600,392,272
GC content (%)	41.51	41.45	41.52	42.30	42.33	42.25	42.55	42.65	42.63	42.65
Complete BUSCOs (%)	99.3	99.3	99.3	99.3	93.4	91.8	94.9	94.8	94.9	95.1
